# Distinctive Subdomains in the Resorbing Surface of Osteoclasts

**DOI:** 10.1371/journal.pone.0060285

**Published:** 2013-03-21

**Authors:** Kinga A. Szewczyk, Karen Fuller, Tim J. Chambers

**Affiliations:** Division of Basic Medical Sciences, St George's, University of London, Cranmer Terrace, Tooting, London, United Kingdom; China Medical University, Taiwan

## Abstract

We employed a novel technique to inspect the substrate-apposed surface of activated osteoclasts, the cells that resorb bone, in the scanning electron microscope. The surface revealed unexpected complexity. At the periphery of the cells were circles and crescents of individual or confluent nodules. These corresponded to the podosomes and actin rings that form a ‘sealing zone’, encircling the resorptive hemivacuole into which protons and enzymes are secreted. Inside these rings and crescents the osteoclast surface was covered with strips and patches of membrane folds, which were flattened against the substrate surface and surrounded by fold-free membrane in which many orifices could be seen. Corresponding regions of folded and fold-free membrane were found by transmission electron microscopy in osteoclasts incubated on bone. We correlated these patterns with the distribution of several proteins crucial to resorption. The strips and patches of membrane folds corresponded in distribution to vacuolar H+-ATPase, and frequently co-localized with F-actin. Cathepsin K localized to F-actin-free foci towards the center of cells with circular actin rings, and at the retreating pole of cells with actin crescents. The chloride/proton antiporter ClC-7 formed a sharply-defined band immediately inside the actin ring, peripheral to vacuolar H+-ATPase. The sealing zone of osteoclasts is permeable to molecules with molecular mass up to 10,000. Therefore, ClC-7 might be distributed at the periphery of the resorptive hemivacuole in order to prevent protons from escaping laterally from the hemivacuole into the sealing zone, where they would dissolve the bone mineral. Since the activation of resorption is attributable to recognition of the αVβ3 ligands bound to bone mineral, such leakage would, by dissolving bone mineral, release the ligands and so terminate resorption. Therefore, ClC-7 might serve not only to provide the counter-ions that enable proton pumping, but also to facilitate resorption by acting as a ‘functional sealing zone’.

## Introduction

The osteoclast is the cell that resorbs bone. It is formed by the differentiation and fusion of mononuclear phagocyte precursors in the presence of macrophage colony-stimulating factor (M-CSF) and receptor activator of NFkB ligand (RANKL) [1], [2]. Bone resorption is an essential component of the process whereby bone undergoes continual removal and replacement throughout life, a process essential to the maintenance of the structural integrity of the skeleton. Excessive or deficient osteoclastic function leads to a number of bone diseases, including osteoporosis and osteopetrosis.

Osteoclasts resorb bone by establishing a circle of close contact between themselves and the bone surface (the ‘sealing zone’). The osteoclast cytoplasm immediately adjacent to the circle of close approach contains abundant F-actin and is devoid of cytoplasmic organelles (the ‘actin ring’, or ‘clear zone’). This circle of close approach is believed to form a peripheral seal, within the confines of which bone resorption occurs. For this, vesicles containing vacuolar H+-ATPase (V-ATPase) and cathepsin K are inserted into the bone-apposed membrane circumscribed by this seal. The surface of the osteoclast within the sealing zone is thrown into convolutions: the ‘ruffled border’. Thus, a ‘resorptive hemivacuole’ is formed between cell and bone, within which protons dissolve the mineral component of bone, and cathepsin K digests the organic matrix [3]. Dissolved products are transported in vesicles from the resorptive hemivacuole and discharged at the opposite, basolateral surface [3], [4]. It has been suggested that these processes of secretion and matrix uptake occur in different domains of the ruffled border [5].

Although the bone-apposed surface is responsible for bone resorption, it has not been accessible to direct visual analysis: our knowledge of this surface is limited to that gained by transmission electron microscopy (TEM) studies.

Recently, we found that ligands for the vitronectin receptor αVβ3 mediate activation of resorptive function in osteoclasts [6]. Thus, while osteoclasts incubated on fibronectin show efficient adhesion and spreading, vitronectin additionally induces osteoclasts to develop actin rings, clear zones and ruffled borders, to develop extracellular digestive activity, and to secrete tartrate-resistant acid phosphatase, as they do on bone. This provides an opportunity to study resorptive activity on surfaces other than bone.

We exploited this opportunity using a novel approach that enables us to inspect the substrate-apposed surface of osteoclasts in the scanning electron microscope (SEM) [6]. For this, glass coverslips are coated with nail-varnish, which is then coated with vitronectin. Osteoclasts are fixed after incubation on this surface. The nail-varnish is then peeled off the glass, inverted onto a glass slide, and gently dissolved in acetone to reveal the undersurface of the osteoclasts.

We have now used this approach to visualize the apical surface of osteoclasts in the SEM after incubation on vitronectin-coated nail-varnish. We then correlated the SEM appearance with the distribution of components crucial to the resorptive machinery of the osteoclast: V-ATPase, which provides the protons that dissolve bone mineral [7], ClC-7, which provides chloride counter-ions necessary for proton pumping by exchanging two chloride ions for one proton [8], and cathepsin K, the enzyme responsible for degradation of the organic matrix of bone. Deficiency of any of these molecules leads to osteopetrosis, a failure of osteoclasts to resorb bone [9], [10], [11], [12], [13].

We found unexpected complexity in the undersurface of activated osteoclasts. Zones of membrane folds correlated with the presence of V-ATPase and F-actin, while cathepsin K was seen in areas of orifice-bearing surfaces. Most interestingly, ClC-7 formed a band immediately inside the actin ring, where, by preventing demineralization of the matrix-attachment site, it might act as a ‘functional sealing zone’.

## Materials and Methods

### Media and reagents

Cells were incubated in minimum essential medium (MEM) with Earle's salts, supplemented with 10% fetal calf serum (FCS), 2 mM glutamine, 100 IU/ml benzylpenicillin and 100 μg/ml streptomycin (all Sigma, Poole, Dorset, UK. Ten % FCS was replaced with 0.1% bovine serum albumin (BSA) (Sigma) for subsequent experiments with vitronectin- or fibronectin-coated substrates. Recombinant human M-CSF and recombinant mouse RANKL were purchased from PeproTech EC (London, UK). Recombinant mouse interleukin-1α (IL-1α) and purified human transforming growth factor-β1 (TGF-β1) were obtained from R & D Systems (Abingdon, Oxon., UK). Immunocytochemical studies were conducted using goat anti-mouse IgG against the B2 subunit of V-ATPase (sc-21210), rabbit polyclonal IgG against the epitope corresponding to amino acids 1-90 (deletion 23–45) mapping at the N-terminus of ClC-7 of human origin (sc-28755), anti-cathepsin K mouse monoclonal antibody (sc-48353), bovine anti-rabbit IgG-rhodamine, donkey anti-goat IgG-FITC, bovine anti-mouse IgG-rhodamine (all Santa Cruz, CA), and goat anti-rabbit IgG-FITC (ABD Serotec, UK). All other reagents were provided by Sigma, unless otherwise stated. Incubations were performed at 37°C in 5% CO_2_ in humidified air.

### Preparation of nail-varnish-coated substrates

A film of clear nail-varnish (Rimmel, London, UK) was spread over the surface of 13 mm glass coverslips using a Pasteur pipette and allowed to dry. To coat these, or non-coated glass coverslips, or 6-well plate wells (Greiner Bio-One, Stonehouse, Gloucestershire), vitronectin or fibronectin (both bovine) was dissolved in water (30 µg/ml). Three hundred µl was placed on coverslips, or 1.5 ml added to 6-well plate wells. Substrates were dried overnight at room temperature in a tissue culture hood.

### Preparation of osteoclast suspensions

MF1 mice (4–8 weeks old) were killed by cervical dislocation. Femora and tibiae were aseptically removed and dissected free of adherent soft tissue. The bone ends were removed and the marrow cavity flushed out into a Petri dish by slowly injecting PBS at one end of the bone using a sterile 25-gauge needle. The bone marrow suspension was passed repeatedly through a 21-gauge needle to obtain a single cell suspension. Bone marrow cells were then washed, resuspended in MEM/FCS and incubated at a density of 3×10^5^ cells/ml for 24 h in a 175-cm^2^ flask (Greiner Bio-One) with M-CSF (5 ng/ml), to deplete the cell preparations of stromal cells. Non-adherent cells were collected by centrifugation and added to 90 mm diameter cell culture dishes (Greiner Bio-One) in MEM/FCS, containing M-CSF (50 ng/ml), RANKL (30 ng/ml) and TGF-β (0.1 ng/ml) (7.2×10^6^ cells in 25 ml for each dish). Cultures were incubated for 5 d, by which time osteoclast formation was maximal. Cells were fed every 2–3 d by replacing 15 ml of culture medium with an equal volume of fresh medium and cytokines.

After formation of osteoclasts on the base of a 90 mm-diameter plastic tissue culture dish, osteoclasts were scraped from the dish into suspension as previously described [14]. To do this, the medium was removed and the cell layer washed 3 times with PBS without calcium and magnesium. Six ml of 0.02% EDTA was added to the dish and cells incubated for 20 min at room temperature. The EDTA was then removed from the dish and replaced with 3.6 ml of calcium/magnesium-free PBS. A cell scraper (Greiner Bio-One) was used to scrape the cells into the PBS, and the resulting cell suspension was agitated using a pipette to ensure uniform cell dispersal, and added to cultures as described below.

### Assessment of osteoclast morphology in the SEM

The technique has been described previously [6]. Briefly, nail-varnish-coated coverslips that had been coated with vitronectin or fibronectin were placed in 24-well plate wells. Osteoclast suspension was added as above and incubated for 5 h. The coverslips were then washed and fixed in 4% glutaraldehyde in 0.2 M sodium cacodylate buffer for 18 h. After fixation the circle of nail-varnish could readily be detached from the glass coverslip, and it was inverted and placed on a glass microscope slide. The nail-varnish was dissolved and the cells dehydrated in an acetone series (30–100%). Care was taken to avoid allowing the circle to dry out. After 5 min in 100% acetone, hexamethyldisilizane (HMDS) was added carefully to the cells, and after a further 5 min the slide was drained and allowed to dry. The glass slide was cut to a square, attached to a stub, sputter-coated with gold and examined in a Cambridge Stereoscan 360 SEM (Cambridge Instrument Company, Cambridge, UK).

### Assessment of osteoclast morphology in the TEM

Slices of bovine cortical bone were prepared as previously described [15]. Osteoclast cell suspension was added to the wells of 24-well plates, each well containing a slice of devitalized cortical bone. The cells were allowed to sediment for 30 min, washed, and incubated in M-CSF (50 ng/ml), RANKL (30 ng/ml) and IL-1α (10 ng/ml) for 24 h.

After incubation, cells were fixed in 4% glutaraldehyde in 0.2 M cacodylate buffer for 18 h, and then processed for TEM.

### Analysis by confocal microscopy

Vitronectin-coated glass coverslips (5 mm diameter), or bone slices were placed in the wells of a 96-well plate (Greiner Bio-One) containing 75 µl MEM/BSA or MEM/FCS respectively. Seventy five µl of the osteoclast suspension was added to each well. Cells were allowed to sediment for 20 min at 37°C before the substrates were washed and transferred to fresh 96-well plate wells and incubated for 5 h in 100 µl MEM/BSA or MEM/FCS with M-CSF (50 ng/ml), RANKL (30 ng/ml) and IL-1α (10 ng/ml).

After incubation, the cultures were fixed in 4% paraformaldehyde in PBS and cells permeabilized with 0.1% Triton X-100 in PBS for 15 min. Cells were then stained for F-actin by incubation in 1 µg/ml FITC-, TRITC-, or Atto 390-conjugated phalloidin for 45 min at 37°C. Immunofluorescence was by standard techniques. The preparations were then washed and mounted in VECTASHIELD mounting medium with DAPI (Vector Laboratories, Peterborough, UK). Cells were inspected in a Zeiss LSM 510 Meta confocal laser scanning microscope (CLSM) (Carl Zeiss, Welwyn Garden City, Hertfordshire, UK).

### Ethics Statement

In accordance with the United Kingdom Animal (Scientific Procedures) Act of 1986, this study did not require a Home Office project license because no regulated procedures were carried out. Mice were humanely killed at a designated establishment by cervical dislocation, which is an appropriate method under Schedule 1 of the Act.

## Results

**Figure 1:**
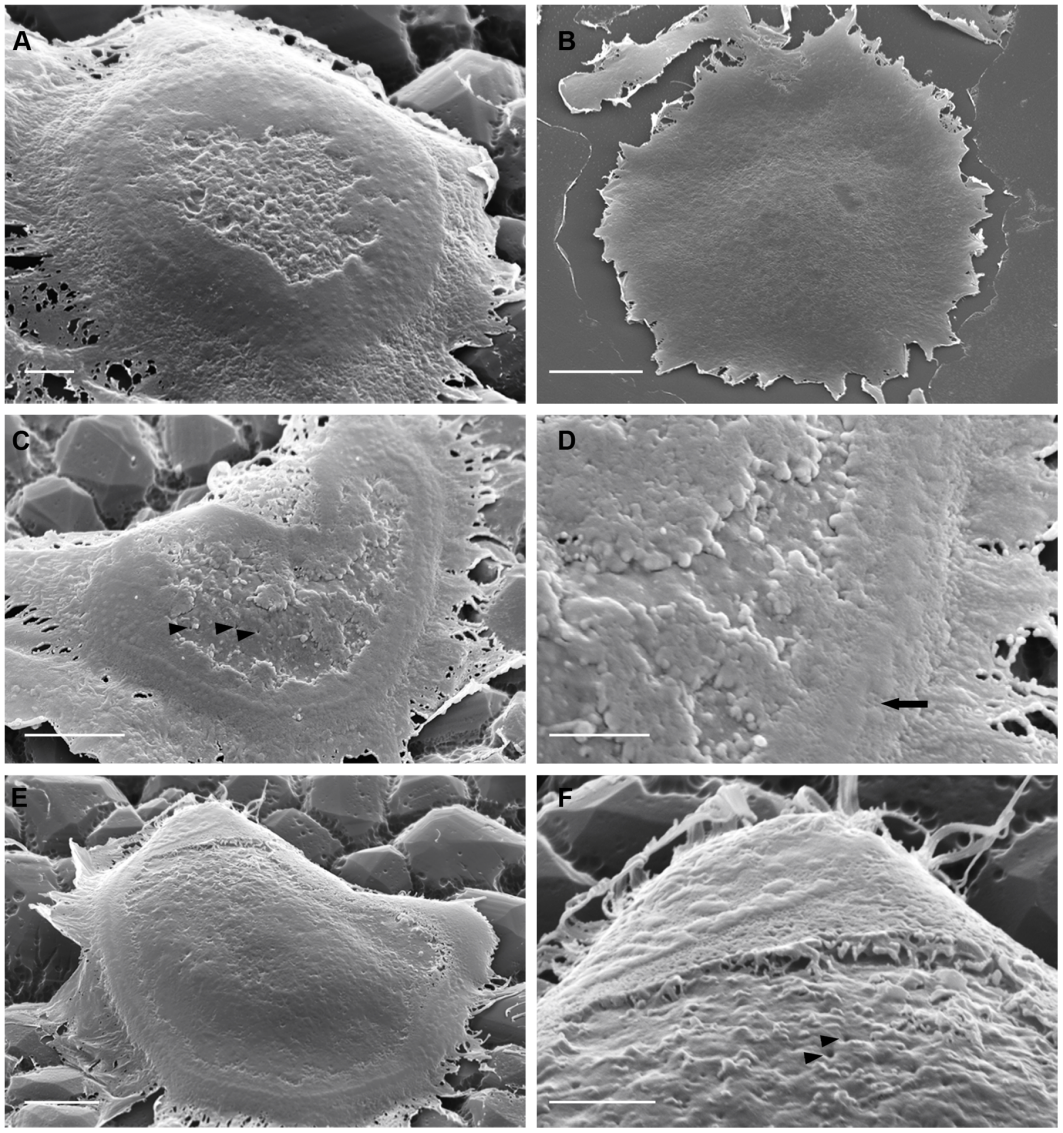
The apical surface of osteoclasts incubated on vitronectin- or fibronectin-coated nail-varnish. Osteoclasts were incubated on coverslips coated with nail-varnish and then vitronectin (A, C–F) or fibronectin (B), fixed, and inverted onto glass slides before removal of nail varnish and preparation for SEM. A: cell incubated on vitronectin, showing a circular arrangement of nodules, which merge in places (at top left of the cell) into ridges. These surround a central region filled with membrane folds. B: substrate-apposed surface of cell incubated on fibronectin lacks these features. C, D: cell shows raised nodules that correspond to podosomes (arrow). Within this podosome crescent are irregular, predominantly peripheral, patches of ruffled membrane. Orifices can be seen in the fold-free surface (arrowheads). E, F: well-spread cell with podosomes predominantly merged into ridges. Folded membrane is flattened against the substrate and is limited to a peripheral strip. Orifices can be seen in the apical membrane central to the peripheral ruffles (F, arrowheads). D and F are magnified portions of C and E, respectively. Bar 2 μm ( A, D, F), 5 μm (C, E) and 10 μm (B).

In-vitro-derived osteoclasts show resorptive behavior on bone and vitronectin-coated substrates within five hours of sedimentation [14]. We found that the periphery of the apical surface of such cells seeded on vitronectin showed peripheral circles or crescents of discrete or confluent nodules (Fig 1). These corresponded in position and organization to the podosomes and actin rings that are commonly observed in F-actin stained preparations of osteoclasts on glass substrates.

Membrane folds were seen inside these peripheral belts. They sometimes covered essentially the whole circumscribed surface (Fig 1A), but were more commonly restricted to a strip immediately inside the podosome ring, together with/without irregular patches surrounded by non-folded membrane (Fig 1C–F). The folds were often flattened against the substrate, but could be identified as folds at the edges of patches and strips, and as crevices in the patches (Fig 1D,F). Orifices could often be seen in areas lacking membrane folds (Fig 1C–F). In contrast, osteoclasts incubated on fibronectin-coated surfaces, which do not induce resorptive behavior [6], showed an undifferentiated appearance, completely lacking in podosomes, membrane folds, and orifices (Fig 1B).

**Figure 2:**
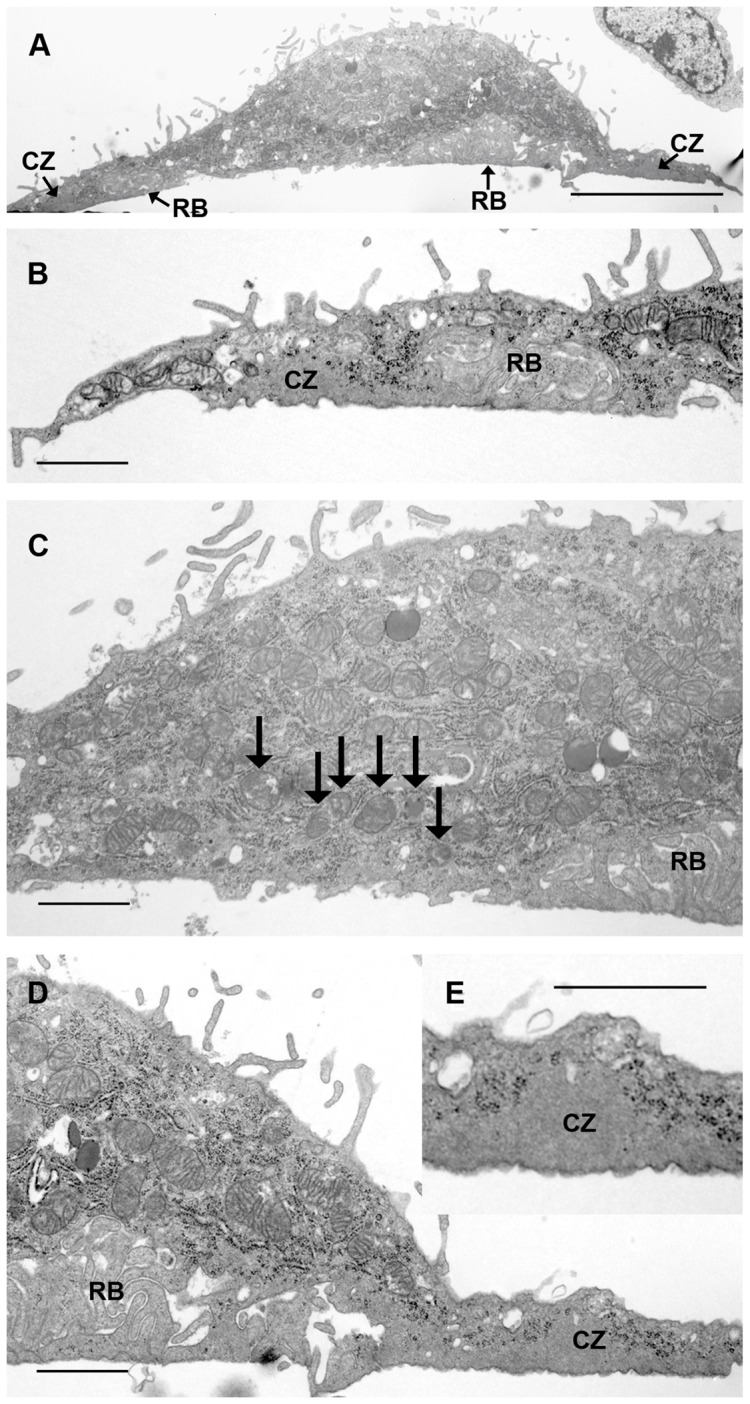
Transmission electron micrographs of osteoclast after incubation on vitronectin-coated tissue culture plastic. A: low magnification of cell shown in B–E. In B, D a peripheral ‘clear zone’ (CZ), clear of organelles, is seen. This is further magnified in D and E, where it is immediately peripheral to a zone of closely-packed membrane folds (ruffled border, RB), which show a pale appearance resembling that of ‘clear zones’. C: central area is devoid of membrane folds. Many vesicles containing electron-dense material (arrows) can also be seen above this area. Bar 5 μm (A) and 1 μm (B–E).

Corresponding appearances were seen after incubation on vitronectin-coated tissue culture dishes, fixed, scraped into suspension and pelleted for TEM (Fig 2): osteoclasts exhibited areas of ruffled border and ruffled-border-free surface. Vesicles were often seen adjacent to the latter. The ruffled border showed pale, rather homogeneous staining characteristics that resembled those of the peripheral ‘clear zone’, or actin ring.

**Figure 3:**
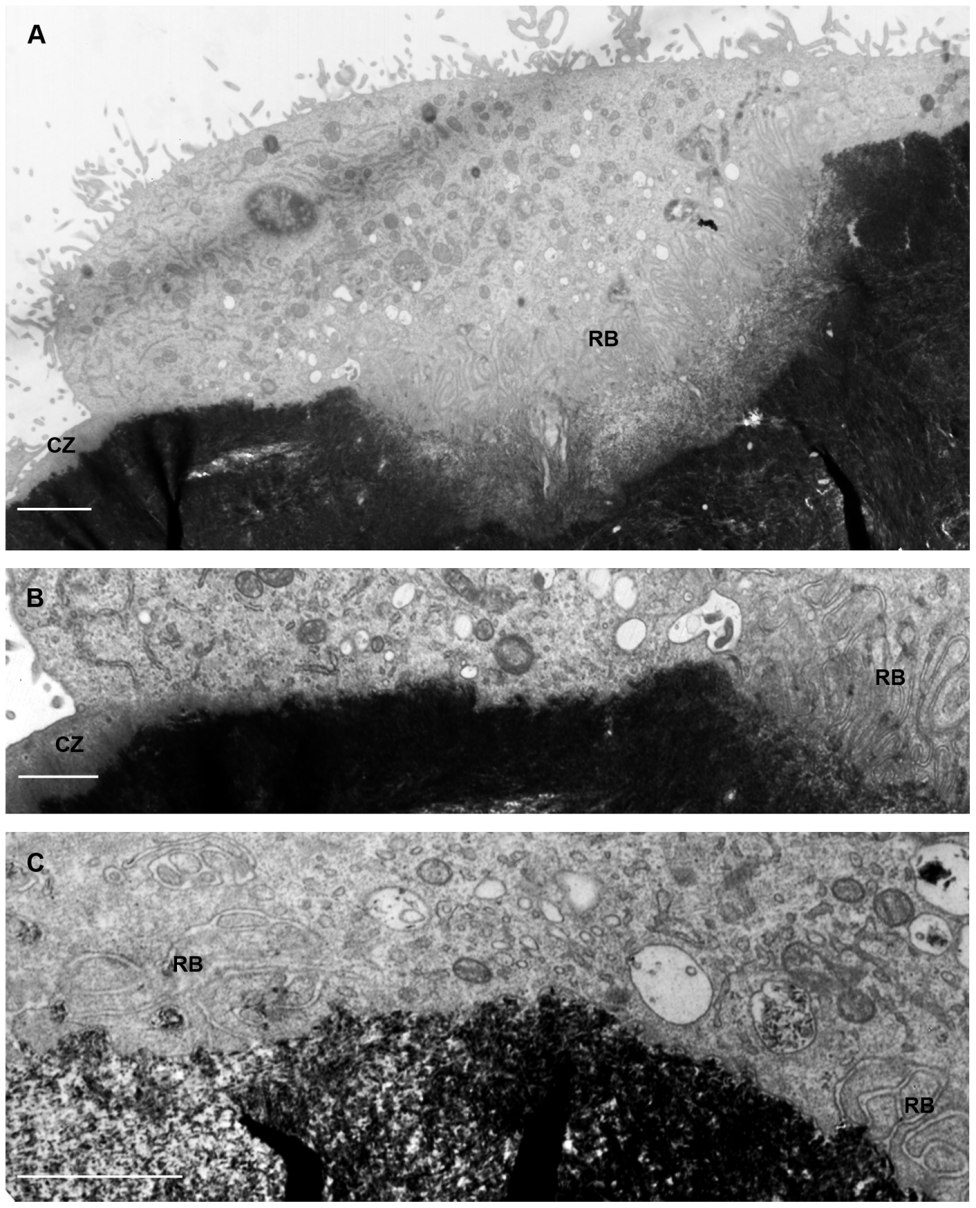
Transmission electron micrographs of osteoclasts after incubation on bone slices. A, B: osteoclast shows clear zone (CZ) at extreme left of field, and extensive ruffled border (RB) to centre and right. Between is an area, shown at higher magnification in B, in which the surface of the cell shows no membrane folds, and in which there is close approach of vesicles to the apical surface. C: view of part of an osteoclast in which zones of membrane ruffles are separated by a region of fold-free membrane. Secondary lysosomes are present just above this fold-free region. Bar 2 µm (A) and 1 µm (B, C).

To determine whether the appearances described above reflect those of osteoclasts resorbing bone, we inspected in the TEM osteoclasts that had been incubated on bone slices. Like those incubated on vitronectin-coated surfaces, osteoclasts on bone frequently showed ruffled border-free surfaces within the confines of the actin ring (Fig 3).

**Figure 4:**
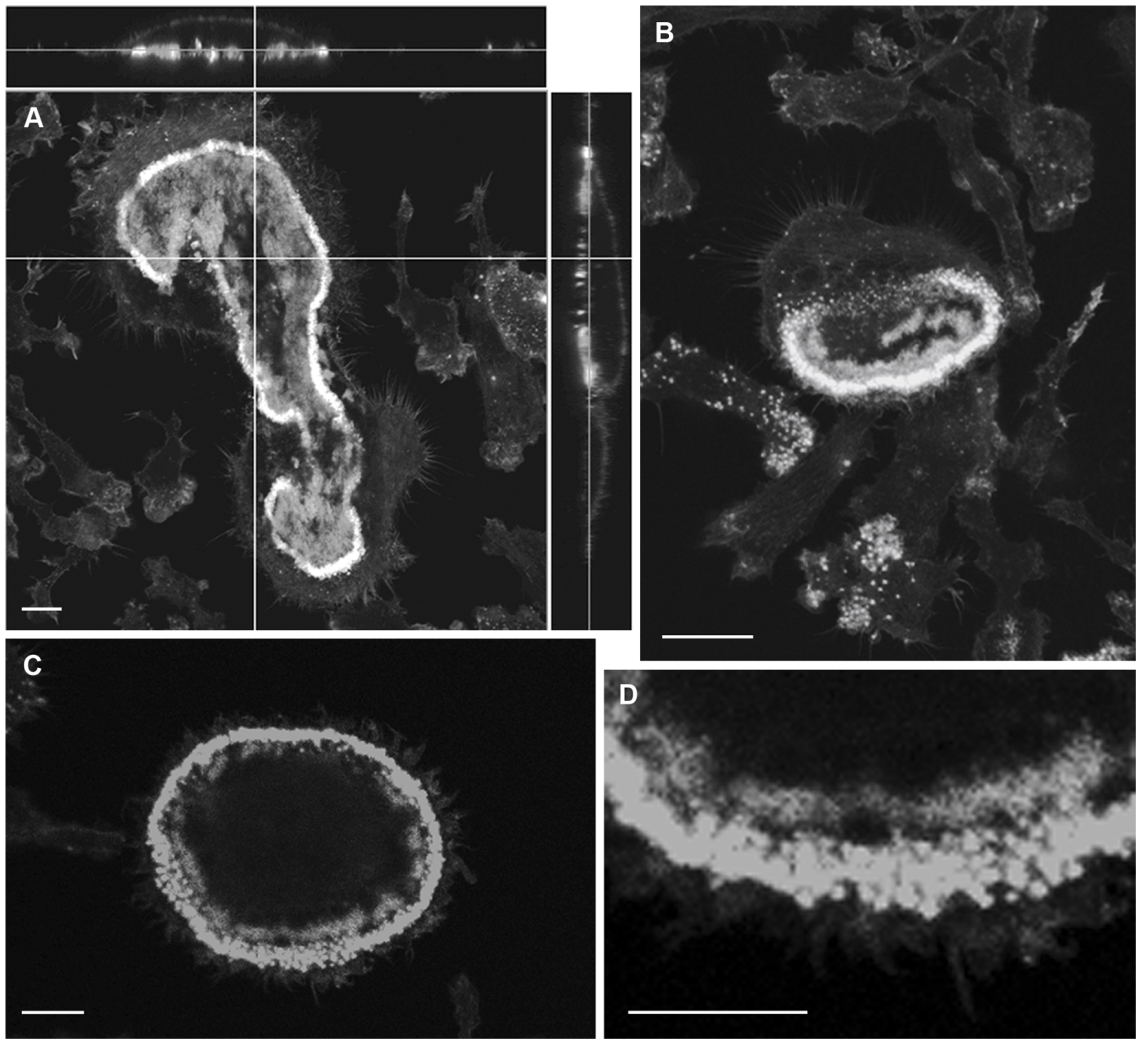
CLSM images of F-actin in osteoclasts after incubation on bone or vitronectin-coated glass coverslips. A: Osteoclast incubated on bone shows strong staining for F-actin in actin ring, but also significant F-actin staining within the ring. The latter is patchy with intervening F-actin-free regions. B: an osteoclast incubated on vitronectin-coated glass coverslip shows a crescent of intensely-F-actin-positive podosomes. F-actin is also present adjacent to the podosome crescent, and as more central patches. C, D: patches of F-actin immediately inside the strongly-positive podosome ring of osteoclast on vitronectin. Bar 10 µm.

Next, we correlated the position of several molecules important to osteoclast function, with the SEM appearances of activated osteoclasts. Actin rings stained strongly on bone, but significant staining was also seen inside the ring, in a patchy distribution (Fig 4A). The z-view shows that this patchy appearance is not accounted for by differences in the plane of section of these confocal images. Similarly, in osteoclasts incubated on vitronectin-coated glass coverslips (Fig 4B–D), patches of F-actin staining were seen within the peripheral F-actin crescents and rings, in a similar distribution to the strips and patches of membrane folds seen in the SEM. This suggests that F-actin is present not only in the actin ring but also the ruffled border. The patchy distribution of F-actin is consistent with the patchy nature of the ruffled border observed in the TEM.

**Figure 5:**
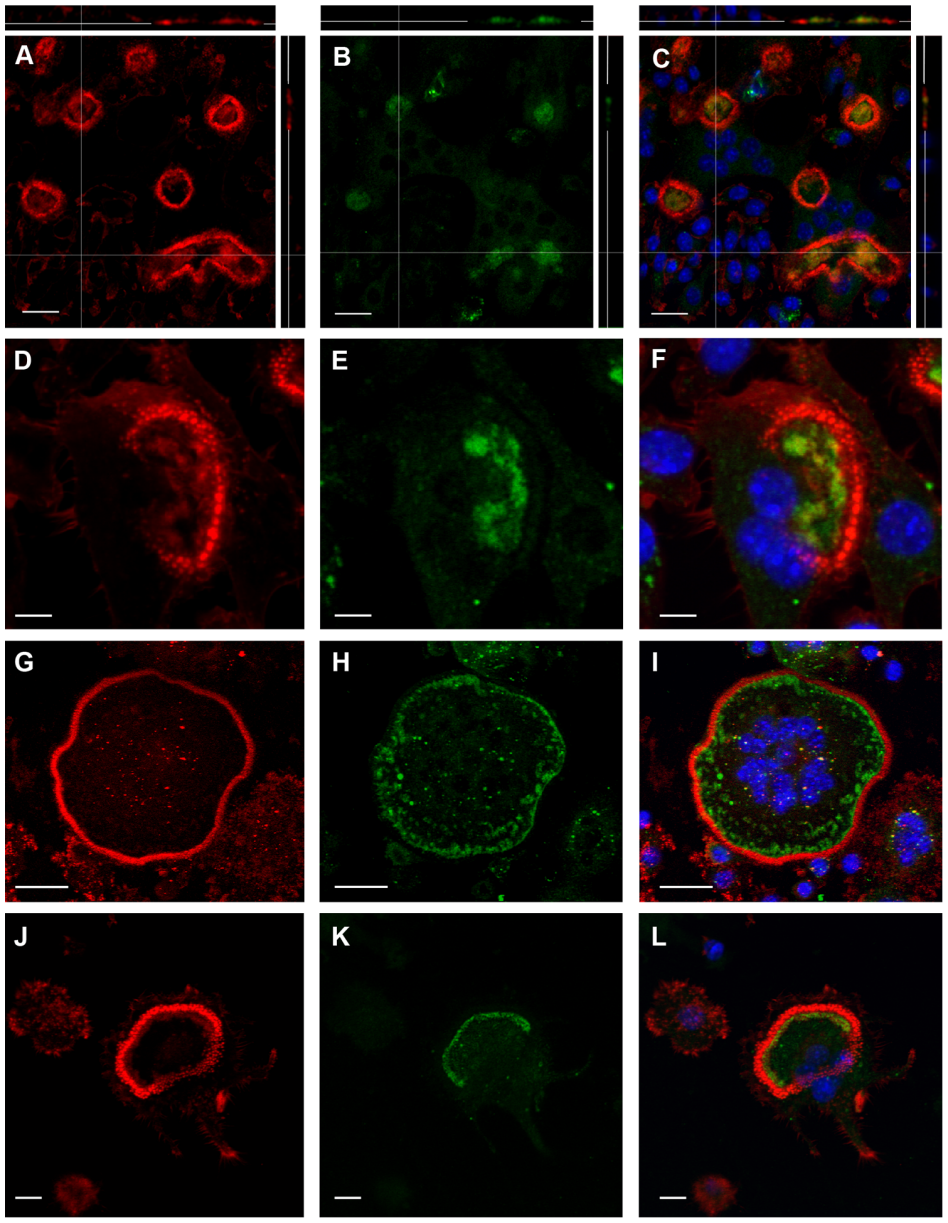
CLSM localization of F-actin (red) and V-ATPase (green) at the apical region of osteoclasts. A–C: In osteoclasts on bone, F-actin (A) and V-ATPase (B) co-localize (C) in a patchy distribution within the F-actin rings. D–L: Like osteoclasts on bone, osteoclasts incubated on vitronectin show prominent F-actin staining in the actin ring/crescent, and noticeable F-actin staining within the ring/crescent. The F-actin within the actin ring tended to represent a greater proportion of smaller (D) than larger diameter (G, J) actin rings. V-ATPase (E, H, K) colocalizes with F-actin (F, I, L,). In all cells the F-actin/V-ATPase is present peripherally and as more central patches. C, F, I, L are counterstained with DAPI. Bar 10 µm (A–C), 5 µm (D–F, J–L) and 25 µm (G–I).

On bone and vitronectin, the distribution of V-ATPase and F-actin was very similar, with frequent co-localization, in the area circumscribed by the peripheral ring/crescent of F-actin (Fig 5). Like F-actin, V-ATPase occurs either as a peripheral strip immediately inside the F-actin ring/crescent, or as a peripheral strip with more central patches (Fig 5D–L). The proportion of the area within rings/crescents showing V-ATPase/F-actin was generally inversely proportional to the area of the ring/crescents. The distribution and frequent co-localization suggest that at least a subset of membrane folds seen in the SEM contain both V-ATPase and F-actin.

**Figure 6:**
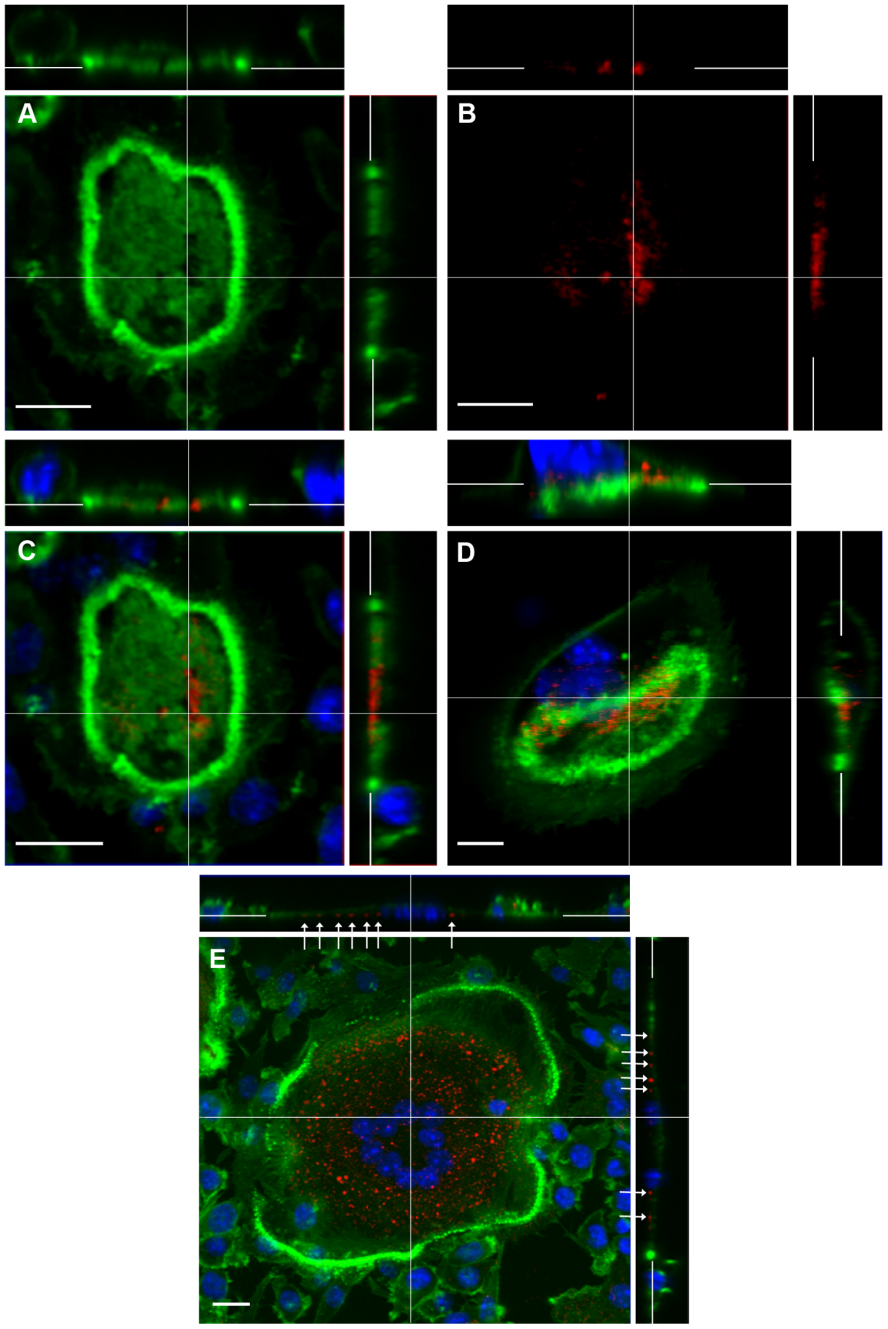
CLSM localization of F-actin (green) and cathepsin K (red) in osteoclasts. A–D: Osteoclasts incubated on bone show patchy distribution of F-actin within the F-actin ring (clearly shown in panel A). In the cell with a non-migratory appearance (A–C), cathepsin K localizes towards the centre of the apex (B), in a region that is free of F-actin (A, C). In the osteoclast showing a migratory morphology (D), cathepsin K localizes to the retracting pole, also in an F-actin-free region (D, z-stacks). E: In osteoclast incubated on vitronectin, cathepsin K localizes to punctuate foci present at the cell-substrate interface (arrows) centrally to F-actin ring. C-E are counterstained with DAPI. Bar 10 µm.

**Figure 7:**
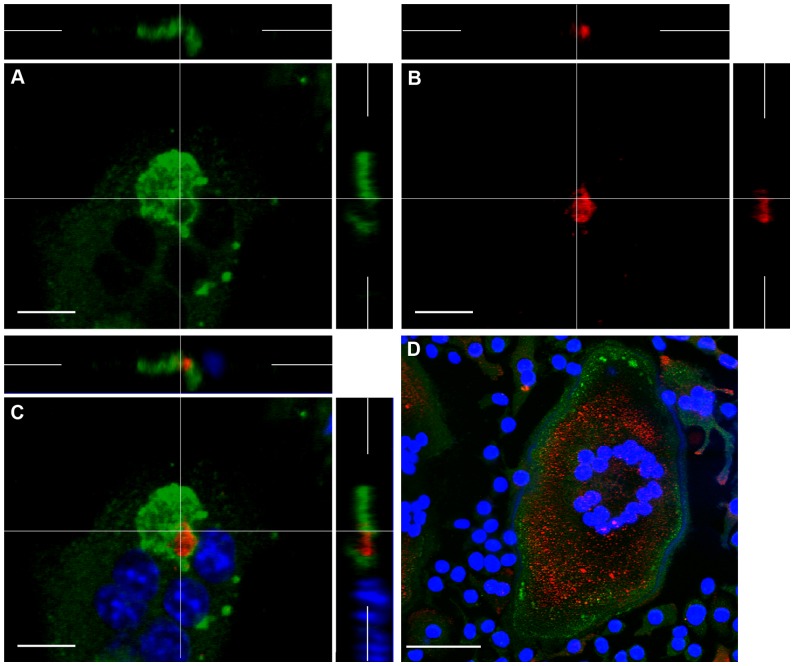
CLSM localization of V-ATPase (green) and cathepsin K (red) in osteoclasts. A–C: Osteoclast incubated on bone shows strong immunolocalizion of V-ATPase in which a patch of cathepsin K immunolocalization is also seen, in a region free of V-ATPase. D: an osteoclast incubated on glass showing cathepsin K immunolocalized in the center of this well-spread cell, while V-ATPase is observed in small patches at the periphery, immediately adjacent to the actin ring (blue). C and D are also counterstained with DAPI to show nuclei. Bar 10 µm.

The distribution of cathepsin K was essentially the inverse of that of actin (Fig 6) and V-ATPase (Fig 7). It was seen in areas within the actin ring where actin and V-ATPase were absent. In cells with crescentic actin rings cathepsin K was seen at the retreating pole of the cell (Fig 6D). Cells on nail-varnish showed a punctate distribution of cathepsin K at the level of the substrate, at the centre of the cell (Fig 6E), in areas associated in the SEM with smooth, orifice-bearing membrane. This punctate staining presumably corresponds to the patches of cathepsin K that have been noted extracellularly, in orifices at the osteoclast-substrate interface, in immunogold TEM preparations of osteoclasts on plastic substrates [16].

**Figure 8:**
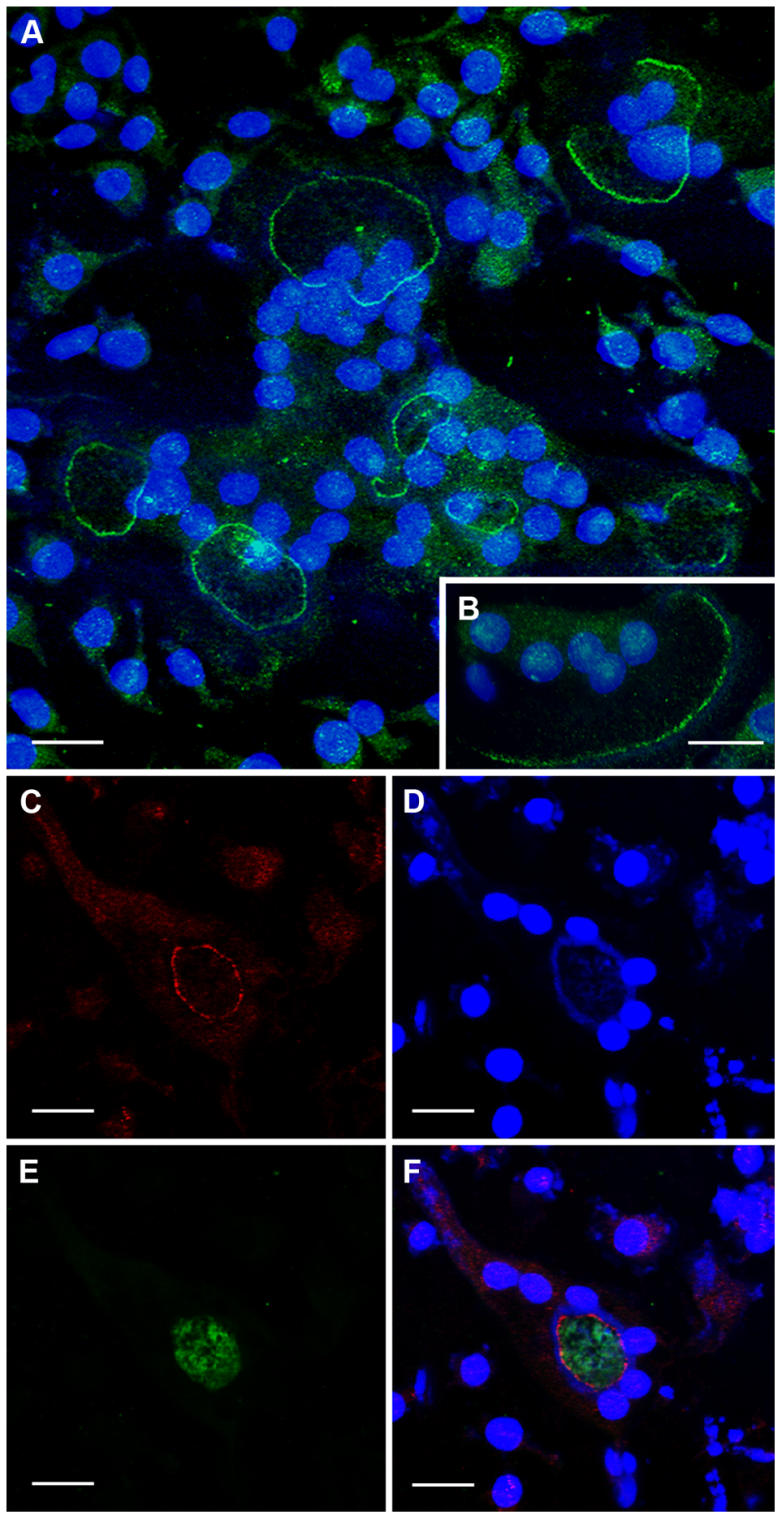
CLSM images showing the distribution of ClC-7, V-ATPase and F-actin in osteoclasts. A, B: In osteoclasts incubated on bone (A) or glass coverslips (B), ClC-7 (green) shows a clear circular distribution. C–F: In osteoclasts incubated on bone, ClC-7 (red) (C) is restricted to a circular strip immediately inside the F-actin ring (D) and immediately outside the central, V-ATPase-rich area (green) (E), and does not co-localize with either V-ATPase or F-actin (F). A, B, D, counterstained with DAPI to show nuclei. Bars 10 µm (A–C) and 50 µm (D).

ClC-7 was immunolocalized as a sharp band immediately apposed to the inner side of F-actin rings and crescents and immediately outside the V-ATPase, as a distinct band showing no co-localization with the latter (Fig 8).

## Discussion

Osteoclasts resorb bone at their substrate-apposed, apical surface, a surface which, despite its importance in bone resorption, has not been accessible to direct visual analysis. We recently developed a novel approach that enables us to inspect the substrate-apposed surface of cells in the SEM [6]. We have now exploited this to visualize the apical surface of activated osteoclasts. To do this, osteoclasts were incubated on vitronectin-coated surfaces, because this activates resorptive behavior: while osteoclasts incubated on fibronectin-coated surfaces show efficient adhesion and spreading, incubation on vitronectin additionally induces osteoclasts to develop many of the characteristics of resorbing cells: actin rings, clear zones and ruffled borders, extracellular digestive activity, and enzyme secretion [6]. We then correlated the SEM observations with the distribution of several components crucial to the resorptive machinery of the osteoclast: V-ATPase, ClC-7, and cathepsin K.

The SEM revealed unexpected complexity in the apical surface of osteoclasts. Dense membrane folds were present in the ruffled border region, but these were typically arranged into peripheral strips, or more central patches of ruffles, surrounded by membrane in which orifices could be seen. This heterogeneity was surprising, because it has been presumed that the ruffled border region of active osteoclasts is a homogeneous region of membrane folds. However, we noted similar, albeit less substantial, regions of flat membrane in the ruffled border region in TEM preparations of osteoclasts on bone. Similar areas can occasionally be seen in published TEM images, but have not to our knowledge been remarked on before [17].

The ‘sealing zone’ was recognizable as a peripheral ring or crescent of nodules which in many places merged to form continuous bands, and which corresponded in F-actin preparations to bands of distinct or confluent podosomes. It is now generally accepted that podosome belts and actin rings are equivalent, and that actin rings result from merging, at higher density or in response to greater stimulation, of individual podosomes [18], [19], [20]. Thus, the reduced podosome density that reveals individual podosomes on plastic might be explained by the greater spreading that occurs in osteoclasts, as in other cells, on smooth substrates [6], [21], [22].

Differences in cell spreading on the smoother surface of the nail-varnish versus bone might explain the patchiness of membrane ruffles: greater spreading might expose onto the osteoclast surface the membrane that lines the cistern-like spaces above the ruffled border of osteoclasts on bone, in a region that is considered to be part of the extracellular space [17], [23], [24]. It is from this surface that partially-digested bone matrix is endocytosed for transcytosis to the basolateral membrane of the cell [17], [18]. It is possible that the orifice-bearing membrane that we have noted in well-spread cells after incubation on vitronectin represents the cisternal region exposed onto the apical surface by cell spreading. This process might account for the greater proportion of V-ATPase- and F-actin-free surface typically seen in well-spread cells (eg, Fig 5). Thus, the greater spreading on smooth artificial substrates provides an opportunity to gain new insights into otherwise-hidden domains of the resorptive surface.

As anticipated, the distribution of V-ATPase correlated with that of the patches and belts of membrane ruffles. F-actin and V-ATPase frequently showed co-localiszation. The implication, that actin can be found in the ruffled border was also reached on the basis of cell-shearing studies [18]. Furthermore, F-actin has been previously noted in the peripheral area of the ruffled border of osteoclasts on bone [5]; and V-ATPase associates with actin [25].

Their high F-actin content suggests that membrane folds can represent an organ of invasion, analogous to lamellipodia or podosomes. Indeed, cell-shearing experiments reported that actin filaments in the ruffled border form a highly interwoven network, similar to that seen in lamellipodia [18]. The protrusive nature of such a structure would explain our observation that the folds appeared to be flattened against the substrate. It has been suggested that the ruffled border of osteoclasts establishes close apposition between cell and bone, such that protons are neutralized by hydroxyapatite with minimal loss by lateral diffusion [26], [27]. A protrusive ruffled border would facilitate the establishment of close apposition, and thereby reduce leakage of protons from a ‘sealing zone’ that has been shown [26] to be permeable even to molecules with molecular mass (*M*r) up to 10,000.

Cathepsin K was immunolocalized to the central area of osteoclasts with a circular actin ring, and towards the retreating pole of osteoclasts with crescents of actin, and showed no co-localization with proton pumps. This distribution corresponds to the distribution of non-folded, orifice-bearing surface between and behind patches and strips of ruffles, and is consistent with an initial demineralization of bone by protons, followed by proteolytic attack of the collagen fibers so exposed. If close apposition of proton-pump-bearing membrane to the bone surface minimizes, through local neutralization, lateral diffusion of protons, the pH in regions containing cathepsin K are likely to be higher than in ruffled-border regions. However, cathepsin K possesses substantial collagenolytic activity even at neutral pH [27].

Electrogenic proton pumping is facilitated by counter-ions. It has been shown that ClC-7, a proton/chloride antiporter that exchanges two chloride ions for one proton is essential for bone resorption [8]. This exchanger showed a characteristic distribution: it formed a sharply-defined band immediately inside actin rings and crescents and peripheral to proton pumps. What can be the explanation for this unexpected pattern?

Since bone owes its ability to activate resorption to avid binding of αVβ3 ligands to bone mineral [6], lateral diffusion of protons would cause mineral dissolution, resulting in release of αVβ3 ligands and termination of resorption. The peripheral localization of ClC-7 means that, while close apposition of the ruffled border to bone targets protons to the mineral surface, any protons that do diffuse laterally will be prevented from escape into the sealing zone by exchange for chloride ions. Such a model, in which mineral at the sealing zone is protected from dissolution by protons, is consistent with the very sharp edge noted in osteoclastic excavations [28], which suggest a degree of localization of protons at odds with the high measured permeability of the sealing zone [26].

The major function of ClC-7 is likely to be the provision of counter-ions to facilitate proton pumping. The evidence for this is that, while osteoclasts do not resorb bone when ClC-7 is completely functionless, osteoclasts that express a form of ClC-7 mutated in such a way that it functions only as a chloride channel, are capable of bone resorption [29]. This shows that the role of ClC-7 in provision of counter-ions is crucial and non-redundant. Thus, despite its peripheral location, ClC-7 is able to act as the sole source of counter-ions for the hemivacuole, presumably via centripetal diffusion of chloride ions and/or centrifugal diffusion of dissolved calcium ions. The latter might facilitate bone resorption by lowering calcium concentrations at the site of mineral dissolution.

However, the excavations formed by osteoclasts expressing the mutated form of ClC-7 mentioned above, that can function only as a channel, appear in published photomicrographs [29] to be of small size. This is consistent with the model suggested above, in which neutralization of peripheral protons maintains bone resorption: the mutated form allows counter-ion transport but does not remove protons at the periphery, so that shortly after the initiation of resorption, the peripheral protons dissolve bone mineral from the sealing zone and lead to early termination of osteoclast activation Thus, ClC-7 might not only supply counter-ions to facilitate proton pumping, but also act as a ‘functional sealing zone’ that prevents access of protons to the sealing zone, thereby maintaining activation of osteoclasts for their resorptive function.
